# Recovery of Lignosulfonates from Spent Sulfite Liquor Using Ceramic Hollow-Fiber Membranes

**DOI:** 10.3390/membranes9040045

**Published:** 2019-03-28

**Authors:** Daniel Humpert, Mehrdad Ebrahimi, Annika Stroh, Peter Czermak

**Affiliations:** 1 Institute of Bioprocess Engineering and Pharmaceutical Technology, University of Applied Sciences Mittelhessen, 35390 Giessen, Germany; daniel.humpert@lse.thm.de (D.H.); mehrdad.ebrahimi@lse.thm.de (M.E.); Annika.stroh@lse.thm.de (A.S.); 2 Department of Chemical Engineering, Kansas State University, Manhattan, KS 66506, USA; 3 Faculty of Biology and Chemistry, Justus-Liebig University of Giessen, 35390 Giessen, Germany

**Keywords:** ceramic hollow-fiber membrane, lignin treatment, lignin fractionation, lignosulfonate, spent sulfite liquor, backflushing

## Abstract

Spent sulfite liquor is an abundant but currently less used wastewater stream from the pulp and paper industry. The recovery of lignin from this resource would provide an inexpensive raw material for the manufacture of fuels and fine chemicals. Here we investigated the suitability of ceramic hollow-fiber membranes for the concentration of spent sulfite liquor as an alternative to common membrane technologies. We tested three ceramic hollow-fiber membranes (3, 8, and 30 nm) in different membrane processes (fed-batch and total recycle mode) and compared their performance with the widely-used tubular membrane geometry. We also evaluated backflushing as a strategy to reduce membrane fouling during filtration. The juxtaposition of the two membrane geometries revealed that wall shear stress is the most important process parameter for the assessment of membrane performance according to permeate flux. The higher the wall shear stress, the higher the permeate flux. Due to the smaller inner diameter of the hollow-fiber membranes, higher wall shear stress can be achieved more easily. Backflushing had no effect on the permeate flux during the concentration experiments.

## 1. Introduction

In 2015, global pulp production amounted to 178.8 million metric tons. In 2016 the European pulp and paper industry consumed 109.0 million metric tons of renewable wood resources, producing 90.9 million metric tons of paper and 37.2 million metric tons of pulp. The majority of the pulp was produced using the kraft or sulfite processes [1,2]. During paper production, lignin must be separated from the raw material (cellulose) because it induces the yellowing of paper products [3]. Regrettably, 99% of the wastewater generated by the pulp and paper industry is incinerated in a recovery boiler to produce energy and to recover coking chemicals [4,5,6]. A major component of this wastewater is lignin. Approximately 49.5 million metric tons of lignin is burned in this way every year [2]. Lignin waste is inexpensive and could be used to manufacture valuable products such as biofuels, vanillin, vanillic acid, synthetic tannins or carbon nanotubes [7,8,9,10]. Accordingly, there is a strong and increasing interest in the development of an economical and environmentally sustainable process to exploit this unused resource.

Membrane filtration offers a unique combination of economy, scalability, and adaptability for the recovery of lignin and the fractionation of wastewater from various industrial processes [11,12,13,14,15]. Ceramic membranes are particularly suitable for this purpose due to the extreme pH and high temperature of the wastewater if it is filtered immediately after removal from the recovery boiler [11,16]. Ceramic membranes are advantageous due to their outstanding chemical, thermal, and mechanical stability [17,18].

Several membrane-bases techniques for the recovery and concentration of lignin containing solutions have been developed over the last decades. Due to the wide dissemination of the kraft process, most of the developed methods use black liquor as the raw material for lignin recovery and only a small part use spent sulfite liquor for the recovery of lignin based structures. Moreover, most studies investigate the usability of organic membranes (e.g. cellulose acetate [19], polysulfone [19], fluoropolymer [19,20] polyethersulfone [20], regenerated cellulose [21]) for the recovery of lignosulfonate. Different membrane geometries (flat disc [19,20,22], tubular [23,24]), as well as different molecular weight cut-offs (1 kDa–0.8 µm [5,19,20,21,22,24,25]) were also well described in the literature. The tubular membranes had inner diameters from 2 to 12,7 mm [5,23,24,25,26]. Nonetheless, ceramic membranes and especially ceramic hollow-fiber membranes are underrepresented in the current literature [11,27].

The most recent generation of innovative ceramic membranes exploit hollow-fiber geometry, which achieves an active layer/volume ratio of up to 9000 m^2^·m^−3^, defined flow conditions, low material costs relative to the active surface area, and low sintering energy and time requirements due to the thinness of the membrane [17,28,29]. Unfortunately, ceramic hollow-fiber membranes are also expensive to manufacture, brittle, and tend to block the flow channel [28]. These combined disadvantages and advantages make it necessary to carry out comprehensive comparative tests of new ceramic hollow-fiber membrane and conventional tubular ceramic membranes.

Here, we evaluated the filtration and concentration of spent sulfite liquor using ceramic hollow-fiber membranes in order to improve our understanding of the filtration process and to compare the filtration performance of different membrane geometries.

## 2. Experimental

### 2.1. Materials

#### 2.1.1. Membranes

Four different ceramic membranes with molecular weight cut-off (MWCO) values of 20 kDa, 30 nm, 8 nm, and 3 nm were evaluated in this study. The hollow-fiber membranes were manufactured by Mann+Hummel GmbH, Ludwigsburg, Germany and the tubular membrane was manufactured by atech innovations GmbH, Gladbeck, Germany. The properties of each membrane are summarized in Table 1.

#### 2.1.2. Spent Sulfite Liquor

The spent sulfite liquor was kindly supplied by Sappi Stockstadt Mill, Stockstadt am Main, Germany, which annually produces up to 445,000 metric tons of fine paper and 145,000 metric tons of pulp [30]. Spent sulfite liquor from the acidic magnesium-based sulfite pulping of beech has a pH of 2.7, a lignosulfonate concentration of 92 g·L^−1^ and was withdrawn before entering the evaporation unit. A detailed analysis of the spent sulfite liquor from a different charge (but the same mill) is presented in Table 2. The composition and the concentration of different spent sulfite liquors from different pulp mills can vary considerably depending on the raw materials and pulping conditions [11].

### 2.2. Methods

#### 2.2.1. Quantification of Lignosulfonate

The lignosulfonate concentration in the permeate and retentate was determined by measuring the absorbance using a BioSpectrometer Basis spectrophotometer (Eppendorf, Hamburg, Germany). The absorbance was measured at 208 nm to minimize absorbance by furfural and hydroxymethylfurfural. Furthermore, the absorbance of lignosulfonate is pH-dependent [31,32]. Therefore, all lignosulfonate samples were diluted in phosphate buffer (0.25 mM potassium dihydrogen phosphate, 0.25 mM disodium hydrogen phosphate, chemicals from Merck, Darmstadt, Germany). Calcium lignosulfonate was used as an external standard. To evaluate membrane performance, the lignosulfonate retention (*R*) was calculated using Equation (1), where *c_p_* and *c_r_* are the lignosulfonate concentrations in the permeate and retentate, respectively.

(1) $$R=\left(1-\frac{c_{p}}{c_{r}}\right)\times 100$$

#### 2.2.2. Zeta Potential

The zeta potential of the ceramic powders (received from the membrane manufacturers) were determined (Zetasizer Nano ZS90, Malvern Panalytical GmbH, Kassel, Gemany) in aqueous suspension by electrophoretic mobility of the powder particles in an electric field. The measurement was repeated three times and the arithmetic mean is represented in the results. The pH-Value was adjusted with hydrochloric acid and sodium hydroxide.

#### 2.2.3. Membrane Filtration

**Figure 1:**
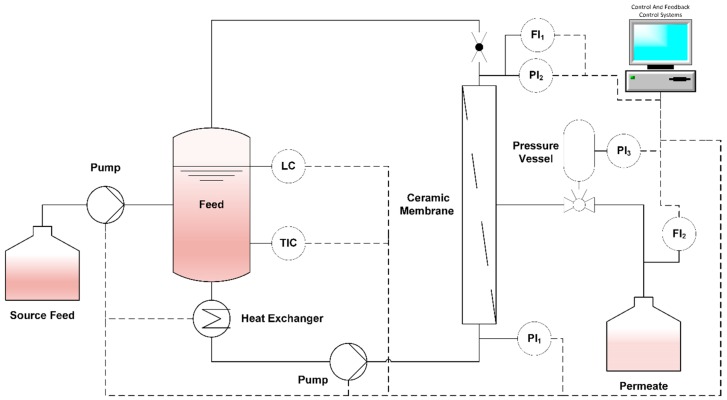
Schematic illustration of the laboratory-scale cross-flow ceramic membrane filtration system. Abbreviations: *L* = level, *T* = temperature, *P* = pressure, *F* = flow, *I* = indicator, *C* = controller.

The laboratory-scale cross-flow ceramic membrane filtration system comprised a 2.5-L feed tank, a variable-volume source feed tank, a rotary vane pump, a heat exchanger, a backflushing system, a control and feedback control system, and the filtration unit (Figure 1). Severe pressure, level, and flow sensors were included to control and record the filtration parameters. All membrane filtration system components in direct contact with the feed were constructed from stainless steel.

The cross-flow ultrafiltration/nanofiltration of the spent sulfite liquor was carried out either in total recycle mode (TRM) or as a fed-batch process with or without backflushing. The feed/retentate temperature was 60 °C, the cross-flow velocity (CFV) was 1–6 m·s^−1^ and the transmembrane pressure (TMP) was 0.5–7.0 bar. The TMP was calculated using Equation (2), where *P_in_* and *P_out_* represent the pressure at the inlet and outlet of the membrane module, respectively [33].

(2) $$TMP=\frac{P_{in}+P_{out}}{2}$$

For the TRM experiments, a feed mass of 1000 g spent sulfite liquor was introduced into the filtration system (both preheated to 60 °C) and the CFV was selected so that the Reynolds number for each membrane geometry was comparable (2827, 5653, 11,307 or 16,960). When the permeate flux became constant, the TMP was increased to the next level, starting at 1 bar and increasing stepwise to 2, 3, 4, 5, and finally 6 bar. The fed-batch experiments were carried out with an initial feed mass of 2.5 kg, again with the feed and filtration system preheated to 60 °C. Unfiltered spent sulfite liquor was added when a volume reduction of approximately 80% was achieved. The fed-batch mode was chosen to investigate the possibility of a continuous filtration. The TMP and CFV were kept constant at 3 bar and 6 m·s^−1^, respectively. During the backflush experiments, the TMP was reversed (values between −2 and −7 bar). The time between backflushes varied from 0.5 to 4.0 h.

#### 2.2.4. Comparison of Different Membrane Geometries

In each experiment, we compared two membranes with similar MWCO values but different inner diameters. When comparing membranes with the same geometry, the filtration parameters (CFV, temperature, and TMP) can be standardized, but this is not possible when comparing different geometries because differences in the inner diameter shift the internal force in relation to the viscous force. The relation between these forces is the Reynolds number [34,35]. Accordingly, the experiments were initially performed with the same Reynolds number, calculated using Equation (3) where ρ is the density, *υ* is the CFV, and *η* is the dynamic viscosity.

(3) $$Re=\frac{\rho \cdot \upsilon \cdot d}{\eta }$$

Moreover we carried out experiments with the same wall shear stress, calculated using Equation (4) where *τ_w_* is the wall shear stress, *ρ* is the density, *v* is the cross-flow velocity, and *λ* is the drag coefficient. The drag coefficient was calculated as a function of the Reynolds number (*Re*), the inner diameter (*d*, and the roughness of the membrane surface (*k*) according to the flow regime [36]. If the flow was laminar (*Re* ≤ 2320) we used Equation (5). If the flow was turbulent and hydraulically smooth (2320 < *Re* < 10^5^) we used Equation (6). In the transition area (65 *d*/*k* < *Re* < 1300 *d*/*k*) we used Equation (7). Finally, if the flow was turbulent and hydraulically rough (*Re* > 1300 *d*/*k*) we used Equation (8).

(4) $$\tau_{w}=\lambda \cdot \rho \cdot \frac{v^{2}}{2}$$

(5) $$\lambda =\frac{64}{Re}$$

(6) $$\frac{1}{\sqrt{\lambda }}=2\cdot log\left(Re\cdot \sqrt{\lambda }\right)-0.8$$

(7) $$\lambda =\frac{1}{{\left[2\cdot log\left(\frac{2.51}{Re\cdot \sqrt{\lambda }}+\frac{0.27}{\frac{d}{k}}\right)\right]}^{2}}$$

(8) $$\frac{1}{\sqrt{\lambda }}=1.74-log\left(\frac{2k}{d}\right)$$

#### 2.2.5. Membrane Cleaning

The ceramic membranes were cleaned before and after every filtration using a 1 wt% solution of the alkaline cleaning agent P3 Ultrasil 14 (Ecolab Deutschland GmbH, Monheim am Rhein, Germany). Cleaning was performed at a feed temperature of 60 °C with a TMP of 1 bar and a CFV of 1 m·s^−1^ for at least 3 h. After cleaning, the membrane was rinsed with pure water and the pure water flux was measured to evaluate the cleaning results.

## 3. Results and Discussion

### 3.1. Zeta Potential

In Table 3, the Zeta-potential values are discussed in combination with the flux results and the fouling behavior in section “Comparison of Membrane Geometries” and “Total Recycle Mode Filtration”. It is important to mention that the measured zeta potential did not reflect the actual potential of the membrane surface during the filtration. The streaming potential would have had to be measured for this purpose. Nonetheless, the measured zeta potential is an indication for the actual surface charge of the membrane surface.

### 3.2. Membrane Filtration

#### 3.2.1. Total Recycle Mode Filtration

The TRM experiments were carried out to evaluate the relationship between the Reynolds number, TMP, lignosulfonate retention, and the critical flux/pressure. The goal was to maximize the lignosulfonate concentration by optimizing lignosulfonate retention with a high permeate flux.

**Figure 2:**
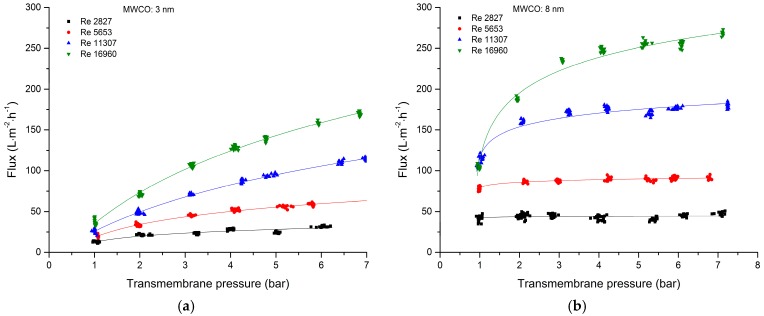

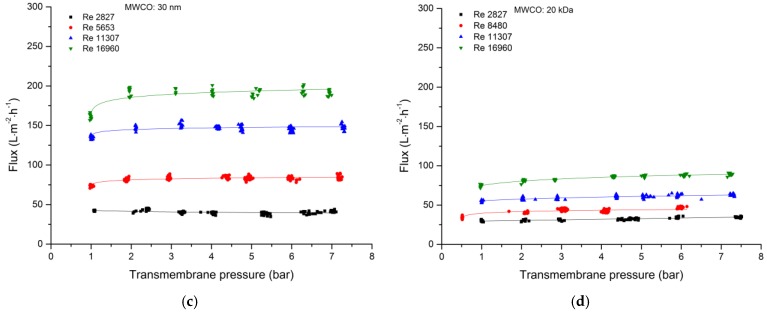
The steady state permeate flux is dependent on the transmembrane pressure of spent sulfite liquor during filtration at different Reynolds numbers. (**a**) 3-nm ceramic hollow fiber membrane. (**b**) 8-nm ceramic hollow fiber membrane. (**c**) 30-nm ceramic hollow fiber membrane. (**d**) 20-kDa tubular ceramic membrane.

The influence of TMP on the permeate flux was investigated at different Reynolds numbers. For all four membranes, the flux increased in a non-linear manner with increasing TMP and tangentially approached the limiting flux (Figure 2). The effect was more pronounced for membranes with a lower MWCO and at higher Reynolds numbers. For the 8-nm membrane, a flux increase with increasing TMP was observed at Reynolds numbers of 11,307 and 16,960. At lower Reynolds numbers, increasing the TMP had no effect on the permeate flux. This effect was also observed for the 20-kDa and 30-nm membranes at Reynolds numbers between 2827 and 16,960. The 20-kDa membrane showed in opposition to the MWCO/pure water flux the lowest permeate flux. This is due to the different wall shear stresses caused by different inner diameters. This behavior is discussed later in “Comparison of Membrane Geometries”. The non-linear relationship with a leveling permeate flux at higher TMPs indicated the formation of a gel layer [22,23,40,41,42]. As soon as the concentration on the membrane surface reaches the gel layer concentration, a homonymous gel layer is formed. At this point, the permeate flux is increasingly dominated by diffusion in the gel layer. An increase in the TMP causes the gel layer to build up and therefore the hydraulic resistance increases [43]. Gel layer formation is also promoted by the positive surface charge of the aluminum oxide/titanium oxide membrane surface and the negative charge of the lignosulfonate molecules. The isoelectric point of aluminum oxide is 8.3–9.5 and that of titanium oxide is 3.9–8.2 [37,44,45]. The strongly acidic pH of the spent sulfite liquor (~2.7) induces a positive zeta potential at the membrane surface, whereas the lignosulfonate molecules are primarily covered by sulfonic groups and a few hydroxyl groups. The zeta potentials of lignosulfonate and aluminum oxide at pH 3 are approximately –43 mV and 38 mV, respectively [37]. This results in the strong adsorption of lignosulfonate to the membrane surface and thereby accelerates the formation of a gel layer [7,22,46]. The zeta potential values from the literature are comparable to the measured zeta potentials (Table 3) of the inner active surface of the membrane and support the argumentation about the fouling behavior of the membrane.

For the 30-nm, 8-nm, and 20-kDa membranes, the limiting flux and limiting pressure were reached within the experimental parameters. It is conspicuous that the limiting flux/pressure was reached later at higher Reynolds numbers and higher MWCO values, which reflects the MWCO-dependent fouling behaviors of ceramic membranes during the filtration of polydisperse mixtures. According to the resistance-in-series model, the loss of flux during filtration involves a combination of pore blocking, adsorption on the membrane surface, concentration polarization, gel layer and cake layer formation [25,47,48,49,50]. A higher MWCO facilitates the permeation of a greater lignosulfonate mass fraction in the active layer. Ultimately, this increases the likelihood of pore blocking, which cannot be prevented by increasing the Reynolds number. Accordingly, increasing the Reynolds number has less effect on the permeate flux, as borne out by our experimental data.

**Figure 3:**
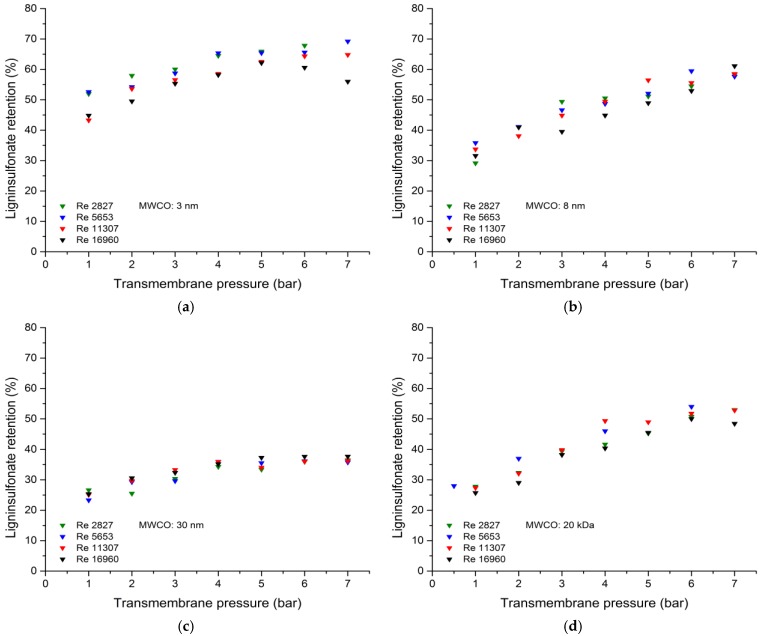
The lignosulfonate retention is dependent on transmembrane pressure during the filtration of spent sulfite liquor at different Reynolds numbers. (**a**) 3-nm ceramic hollow fiber membrane. (**b**) 8-nm ceramic hollow fiber membrane. (**c**) 30-nm ceramic hollow fiber membrane. (**d**) 20-kDa tubular ceramic membrane.

The correlation between the TMP and lignosulfonate retention for all four membranes is shown in Figure 3. As the TMP increased from 1 to 7 bar, the quantity of retained lignosulfonate increased from ~48 to ~63% for the 3-nm membrane, from ~32 to ~59% for the 8-nm membrane, from ~25 to ~37% for the 30-nm membrane, and from ~27 to ~51% for the 20-kDa membrane. In many cases a decrease of the lignosulfonate retention was observed at higher Reynolds numbers and higher transmembrane pressures. This behavior may be caused by different phenomena. First, at high Reynolds numbers and high transmembrane pressures the feed temperature slightly increased (~3 °C) reflecting the high energy input of the rotary vane pump. This leads to higher lignosulfonate solubility and therefore the retention decreased. Second, at the highest transmembrane pressures/Reynolds numbers the flux was at its maximum. This may increase the concentration polarization of the retained lignosulfonate molecules and decrease the lignosulfonate retention [51].

The greater retention reflected the compression of the probably formed gel layer at higher pressures, thus causing more of the spent sulfite liquor to reach the membrane surface. This vicious layer is responsible for the pre-sieving of the lignosulfonate molecules [6,16,42,52]. We found that the Reynolds number made no significant difference to the quantity of retained lignosulfonate.

As expected, the retention decreased as the MWCO of the membranes increased. The lignosulfonate molecules in the spent sulfite liquor have a high degree of polydispersity, and a greater proportion of these lignosulfonate molecules can permeate the membrane as the MWCO increases. The retention performance of the 8-nm hollow-fiber membrane and the 20-kDa tubular ceramic membrane were comparable.

#### 3.2.2. Fed-Batch-Filtration

Fed-batch filtration experiments were carried out to evaluate the correlation between flux loss and lignosulfonate retention during the concentration of spent sulfite liquor. This was achieved by progressively reducing the feed volume (Table 4). Interestingly, the 8-nm hollow-fiber membrane showed the highest initial flux (235 L·m^−2^·h^−1^), followed by the 30-nm membrane (218 L·m^−2^·h^−1^) and the 3-nm membrane (160 L·m^−2^·h^−1^). It is likely that the pore size of the 30-nm membrane combined with the molecular weight distribution of the lignosulfonate molecules promoted rapid pore blocking, leading to the loss of flux more quickly compared to the 8-nm membrane. The lowest initial flux was observed for the 20-kDa tubular membrane (94 L·m^−2^·h^−1^).

When the feed volume was reduced by 30%, the 8-nm membrane achieved the highest flux (184 L·m^−2^·h^−1^) followed by the 30-nm membrane (156 L·m^−2^·h^−1^) and the 3-nm membrane (123 L·m^−2^·h^−1^). The 20-kDa membrane again showed the lowest flux (64 L·m^−2^·h^−1^). The loss of flux at a volume reduction of 30% was therefore lowest for the 3-nm membrane (20%), followed by the 8-nm membrane (22%), the 30-nm membrane (28%) and finally the 20-kDa membrane (32%). A similar trend was observed at a volume reduction of 50% for the three membranes with the lowest MWCO values. The 3-nm membrane showed the lowest decline in flux (34%), followed by the 8-nm membrane (39%) and the 20-kDa membrane (45%). However, in this case the 30-nm membrane (with the highest MWCO) also showed a flux loss of 33%, indicating that fouling was less severe.

Multiple tests were carried out with the feed volume reduced by 80%, resulting in fluxes of <40 L·m^−2^·h^−1^ for the 3-nm membrane, <42 L·m^−2^·h^−1^ for the 8-nm membrane, <97 L·m^−2^·h^−1^ for the 30-nm membrane and <12 L·m^−2^·h^−1^ for the 20-kDa membrane (Table 4). The flux loss due to additional spent sulfite liquor addition is comparatively low. It may be assumed that the flux loss is primary caused by the increase of the viscosity. At a feed volume reduction of 80% the flux decreased for the 3 nm membrane from 40 to 35 L·m^−2^·h^−1^, for the 8 nm membrane from 42 to 34 L·m^−2^·h^−1^, for the 30 nm membrane from 97 to 91 L·m^−2^·h^−1^, and for the 20 kDa membrane from 12 to 11 L·m^−2^·h^−1^. The absolute flux values at 80% volume reduction were particularly interesting. As expected, the highest flux was observed for the membranes with the highest MWCO: ~97 L·m^−2^·h^−1^ for the 30-nm membrane, 12 L·m^−2^·h^−1^ for the 20-kDa membrane, and similar values of ~40 and 42 L·m^−2^·h^−1^ for the 3-nm and 8-nm membranes, respectively. The flux was higher for the 8-nm membrane when the volume reduction was smaller. Therefore, the flux loss due to membrane fouling after a volume reduction of 50% was higher for the 8-nm membrane.

**Figure 4:**
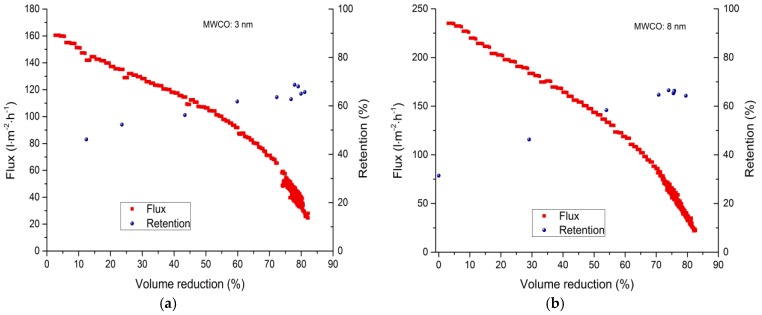

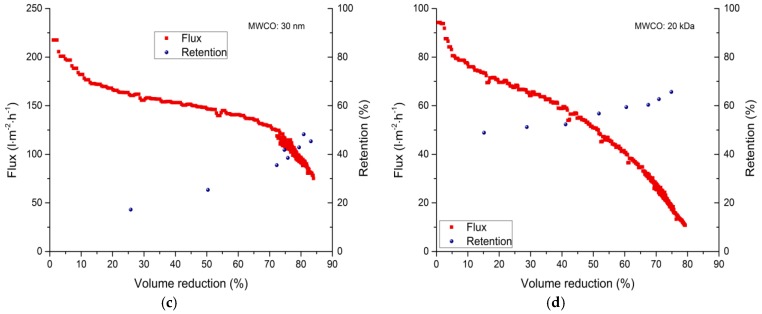
Flux loss and lignosulfonate retention depends on volume reduction during the filtration of spent sulfite liquor. (**a**) 3-nm ceramic hollow fiber membrane. (**b**) 8-nm ceramic hollow fiber membrane. (**c**) 30-nm ceramic hollow fiber membrane. (**d**) 20-kDa ceramic tubular membrane. Process parameters: *Re* = 12,720, transmembrane pressure = 3 bar, and temperature = 60 °C.

The lignosulfonate retention and permeate flux characteristics of each membrane after volume reduction are summarized in Figure 4. As expected, lignosulfonate retention increased in line with volume reduction [5,6]. For the 3-nm membrane, the retention increased from 46 to 68%, for the 8-nm membrane the increase was 46 to 66%, for the 30-nm membrane the increase was 17 to 48%, and for the 20-kDa membrane the increase was 48 to 65%. The higher retention was caused by the buildup of a cake layer on the membrane surface. This cake layer works as an additional filter and enhances rejection by preventing smaller lignosulfonate molecule from passing through the membrane [5,7,49,53].

### 3.3. Comparison of Membrane Geometries

Next, we compared membrane geometries with different inner diameters, focusing on the 8-nm hollow-fiber membrane and 20-kDa tubular ceramic membrane because of their similar MWCO values (based on manufacturers’ specifications and the retention data acquired in our TRM experiments). Only the flux values differed substantially in the TRM experiments, with the 8-nm hollow-fiber membrane showing significantly higher fluxes than the 20-kDa tubular membrane under the same process parameters. The comparable lignosulfonate retention at different fluxes may reflect the different pore size distributions of the two membranes. Alternatively, the smaller inner diameter of the hollow-fiber membrane may induce higher wall shear stress that inhibits the formation of a fouling layer on the membrane surface. To evaluate the effect of wall shear stress on membrane performance, we conducted TRM experiments with standardized wall shear stress. We maintained the same process parameters as described above for the other TRM experiments except the CFV, which was modified to ensure the same wall shear stress for each membrane.

**Figure 5:**
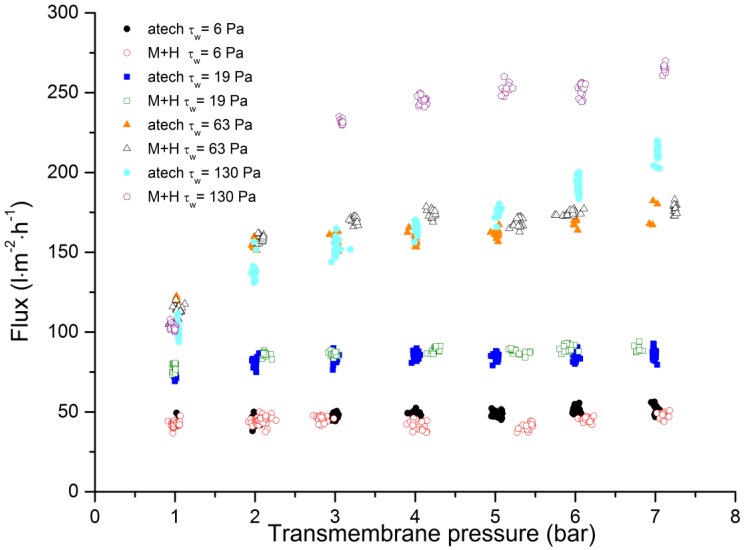
The relationship between transmembrane pressure and permeate flux for the 20-kDa tubular ceramic membrane and the 8-nm hollow-fiber membrane during the filtration of spent sulfite liquor.

We found that permeate flux was dependent on the TMP, and that both membranes performed similarly except at the highest wall shear stress value (Figure 5). Increasing the wall shear stress generally increased the flux by inhibiting the formation of a fouling layer on the membrane surface [54,55,56,57]. As the wall shear stress levels increased beyond 63 Pa, the flux continued to increase with TMP for the hollow-fiber membrane but not the tubular membrane. The flux increased at a wall shear stress of 130 Pa (TMP = 7 bar) for the 20-kDa membrane was induced by an increase in the feed temperature, reflecting the high energy input of the rotary vane pump. The flux of the tubular membrane at a wall shear stress of 130 Pa was the same as at 63 Pa. This behavior should be the subject of subsequent studies.

The flux differences at wall shear stresses exceeding 63 Pa may reflect the different production methods for the two ceramic membranes. Hollow-fiber membranes are produced by spinning, and the membrane structure is formed by phase inversion, which results in a finger-like pore structure in the support layer and a sponge-like pore structure in the separation layer. In contrast, tubular ceramic membranes are produced by dip-coating and have a consistent sponge-like pore structure. The inner surface area is generally higher for membranes with a sponge-like pore structure, resulting in more pronounced adsorption effects and a greater likelihood of fouling [58,59,60,61,62,63,64]. Our data show that the wall shear stress is an important parameter for the evaluation of membrane performance in terms of the permeate flux, especially when comparing different membrane geometries.

**Figure 6:**
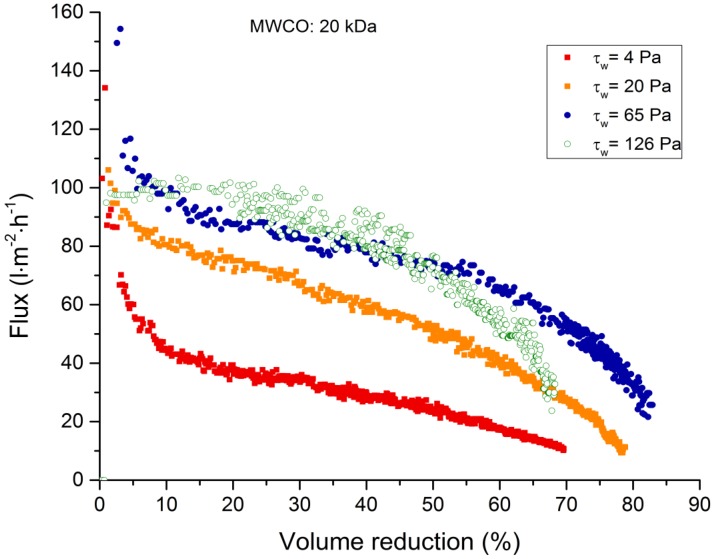
Flux loss is dependent on volume reduction during the filtration of spent sulfite liquor using a 20-kDa tubular ceramic membrane at a transmembrane pressure of 3 bar, a feed temperature of 60 °C, and at wall shear stresses of 4–126 Pa.

To confirm the flux behavior of the 20-kDa tubular ceramic membrane when the wall shear stress exceeds 63 Pa, batch filtration experiments were conducted at wall shear stresses of 4–126 Pa while maintaining a fixed feed temperature of 60 °C and a TMP of 3 bar (Figure 6). As indicated by the TRM experiments, increasing the wall shear stress caused an increase in permeate flux even in batch-filtration experiments. Furthermore, no increase in permeate flux was observed for wall shear stresses greater than 65 Pa, resulting in comparable values at 65 and 126 Pa. Our data suggest that flux behavior in batch filtration experiments can be predicted based on the outcome of TRM experiments. However, we observed an important difference in terms of flux development at wall shear stresses of 65 and 126 Pa. When the volume is reduced by 50%, the flux drops more rapidly at a wall shear stress of 126 Pa compared to 65 Pa. Due to the high wall shear stress, the thickness of the fouling layer is reduced making the membrane surface more accessible and thus more susceptible to pore blocking.

### 3.4. Backflushing

**Figure 7:**
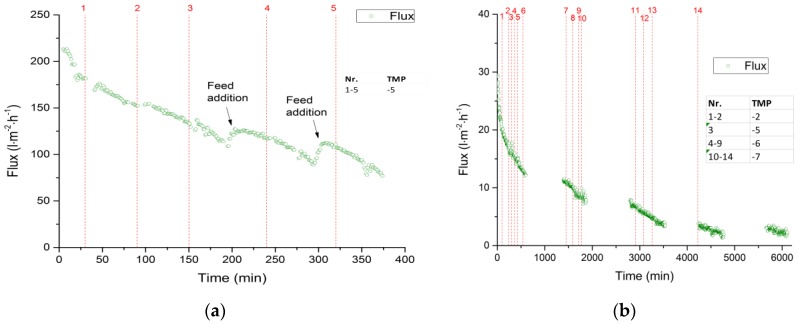
Membrane filtration performance during the filtration of spent sulfite liquor (feed temperature = 60 °C, *CFV* = 6 m·s^−1^, *Re* = 12,876, *τ_w_* = 140 Pa, *TMP* = 3 bar). (**a**) 30-nm ceramic hollow fiber membrane. (**b**) 3-nm ceramic hollow fiber membrane (data gaps show where the filtration process was interrupted overnight).

The permeate flux of the 3-nm and 30-nm hollow-fiber membranes were measured after filtration for durations of 6100 and 375 min, respectively. The flux decline during this time is shown in Figure 7. Both filtrations were carried out at a feed temperature of 60 °C, with a CFV of 6 m·s^−1^, a Reynolds number of 12,876, a wall shear stress of 140 Pa, and a TMP of 3 bar. To evaluate the effect of backflushing as a strategy to minimize membrane fouling and thus the loss of flux, we compared backflushing for 5 s at TMPs ranging from −5 to −7 bar. We found that backflushing had no effect on the permeate flux through the 30-nm and 3-nm membranes, indicating that inverting the permeate flux had no measurable impact on membrane fouling. The strong absorption capacity of the fouling layer, reflecting the substantial difference in zeta potential between the membrane surface and lignosulfonate molecules, cannot be surmounted by the force of the reverse-flowing spent sulfite liquor permeate in the membranes we tested. However, backflushing with permeate was shown to be an effective strategy to prevent membrane fouling during the filtration of bleaching effluent [65,66,67]. Further studies are therefore required to evaluate the effect of the magnitude of zeta potential differences on membrane fouling and the backflushing cleaning effort.

### 3.5. Membrane Cleaning

Due to the high investment costs of ceramic hollow-fiber membranes, a successful and efficient cleaning strategy is of great significance. Within the conducted experiments, irreversible membrane fouling was not of great importance for the investigated membranes. Overall, the pure water flux was, at its worst, 95% of the initial pure water flux. Nevertheless, it can be assumed, that irreversible fouling is of great importance if the membrane is used over a long period [41,68].

## 4. Conclusions

We have shown that it is possible to concentrate lignosulfonate from spent sulfite liquor using ceramic hollow-fiber membranes. TRM and fed-batch experiments revealed that an increase in the Reynolds number/wall shear stress leads to an increase of the permeate flux. Furthermore, lignosulfonate retention increased with increasing TMP, volume reduction, and with decreasing MWCO. The 3-nm ceramic hollow-fiber membrane achieved the highest lignosulfonate retention (up to 69%), and at a volume reduction of 80% the flux loss was lower than that of the 8-nm ceramic hollow-fiber membrane. The flux loss during concentration is mainly caused by an increase in feed viscosity.

The comparison of the 8-nm ceramic hollow-fiber membrane and the 20-kDa tubular ceramic membrane showed that wall shear stress is an important process parameter for the comparison of membranes with different geometries. Moreover, the hollow-fiber membrane showed a significant increase in flux compared to the tubular membrane when the wall shear stress exceeded 63 Pa.

In conclusion, ceramic hollow-fiber membranes are comparable with or even better than common tubular ceramic membranes for the concentration of spent sulfite liquor. The hollow-fiber membranes achieved comparable lignosulfonate retention and higher permeate flux when the wall shear stress was increased. Moreover, hollow-fiber membranes have a higher ratio of active membrane surface to membrane volume. Consequently, operation at a given wall shear stress is more energy-efficient and therefore less expensive for ceramic hollow-fiber membranes compared to tubular membranes with a larger inner diameter.

## Figures and Tables

**Table 1:** Properties of the ceramic membranes compared in this investigation.

Cut-Off	Manufacturer	Material Active Layer Inner Surface	Length (m)	Inner Diameter (mm)	Membrane Geometry	Membrane Area (m^2^)	Pure Water Flux (L·m^−2^·h^−1^·bar^−1^)
**20 kDa**	atech innovations	Al_2_O_3_	0.25	6	Tubular	0.0047	262
**3 nm**	Mann+Hummel	TiO_2_	0.25	2	Hollow fiber	0.0038	49
**8 nm**	Mann+Hummel	TiO_2_	0.25	2	Hollow fiber	0.0030	251
**30 nm**	Mann+Hummel	Al_2_O_3_	0.25	2	Hollow fiber	0.0038	563

**Table 2:** Characterization of spent sulfite liquor.

Component	Value
Density (20 °C)	1060 kg·m^−3^
pH (20 °C)	2.7
Dry residue (after 72 h at 105 °C)	12.5%
Ignition loss (550 °C)	88.8%
Chemical oxygen demand	138.3 mg·kg^−1^
Glucose	0.39 g·kg^−1^
Xylose	11.48 g·kg^−1^
Galactose and rhamnose	1.02 g·kg^−1^
Arabinose	0.20 g·kg^−1^
Mannose	0.56 g·kg^−1^
Methanol	320 mg·kg^−1^
Ethanol	50 mg·kg^−1^
Hydroxymethylfurfural	24 mg·kg^−1^
Acetic acid	9.0 g·kg^−1^

**Table 3:** Zeta potential of the inner membrane surfaces at pH 2 [37,38,39].

Material	Zeta Potential	
	Measured	Literature
Al_2_O_3_	(33.9 ± 7.3) mV	~38 mV
TiO_2_	(26.6 ± 5.3) mV	~30 mV

**Table 4:** The flux through ceramic membranes given a feed volume reduction (VR) of 0%, 30%, 50% or 80%. Process parameters: *Re* = 12,720, transmembrane pressure = 3 bar, and temperature = 60 °C. The fluxes region at a VR of 80% results from different VR at one fed-batch experiment. Due to the experimental setup it was not possible to achieve at every experiment a VR of 80% within the first VR.

Membrane	0% VR (L·m^−2^·h^−1^)	30% VR (L·m^−2^·h^−1^)	50% VR (L·m^−2^·h^−1^)	80% VR (L·m^−2^·h^−1^)
3 nm	160	128	106	40–35
8 nm	235	184	144	42–34
30 nm	218	156	147	97–91
20 kDa	94	64	51	12–11
